# Origin-Based Social Distance Among Nurses in Croatia: Implications for Migrant-Sensitive Healthcare Delivery

**DOI:** 10.3390/healthcare14213581

**Published:** 2026-10-23

**Authors:** Aleksandar Racz, Andrea Armano, Ljerka Armano

**Affiliations:** 1 Faculty of Health Studies, University of Zagreb, Mlinarska cesta 38, 10000 Zagreb, Croatia; ljerka.armano@kbcsm.hr; 2 Faculty of Health Studies, University of Rijeka, Viktora Cara Emina 5, 51000 Rijeka, Croatia; 3 Faculty of Medicine, University of Rijeka, Braće Branchetta 20/1, 51000 Rijeka, Croatia; 4 Aspira University of Applied Sciences, Mike Tripala 6, 21000 Split, Croatia; andrea.armano@aspira.hr; 5 Department for Prevention of Hospital Infection, University Hospital Centre Sestre Milosrdnice, Vinogradska cesta 29, 10000 Zagreb, Croatia; 6 Department of Nursing, University of Applied Sciences Ivanić-Grad, Moslavačka ulica 13, 10310 Ivanić-Grad, Croatia

**Keywords:** nurses, migrant-sensitive healthcare, healthcare workforce, social distance, healthcare equity, quality of care, intercultural contact

## Abstract

**Background and Objectives:** Migrant-sensitive healthcare depends on organisational communication support and on a workforce prepared to provide equitable care across perceived social and cultural differences. This study treated origin-based social distance as a workforce-context measure, rather than a measure of clinical conduct. It examined whether it varied across migrant-worker labels and which identity-, threat-, and contact-related factors were associated with it. **Materials and Methods:** An anonymous cross-sectional online survey was completed by 453 qualified nurses and nursing technicians in Croatia between March and April 2026. Participants rated the closest relationship they would accept with Croats and with Filipino, Nepalese, Indian, and Bangladeshi workers and migrants from Arab countries using an adapted seven-level Bogardus measure. Repeated-measures analysis, Bonferroni-adjusted comparisons, correlations, and hierarchical regression were used. **Results:** Social-distance ratings differed across the five migrant-worker labels (F(4, 1808) = 162.75, *p* < 0.001, partial η^2^ = 0.265). Distance was lowest toward Filipino workers and highest toward migrants from Arab countries. National exclusiveness (β = 0.29), economic insecurity and status threat (β = 0.21), and ethnocentrism (β = 0.20) were associated with greater distance. In contrast, contact quality (β = −0.19) and wider travel and intercultural experience (β = −0.13) were associated with lower distance. **Conclusions:** The findings identify a potentially modifiable workforce context for migrant-sensitive care and support organisation-wide safeguards for equitable communication and continuity. They do not demonstrate unequal clinical conduct or patient harm; those pathways require direct prospective evaluation using care-process, patient-experience, safety, and health-outcome measures.

## 1. Introduction

International migration is a routine part of contemporary healthcare. Migrants use services as patients, accompany family members, work in health and social care, and participate in local communities. Their access to safe care is shaped by formal entitlement, language, health literacy, prior experiences, and the organisation of services [1,2,3,4,5,6,7]. For nurses, these conditions affect ordinary clinical work: assessment, explanation, consent, reassurance, treatment instructions, discharge planning, and advocacy.

Education in cultural competence is often proposed as a response to growing diversity, but recent nursing and European reviews show that knowledge of group customs is not enough. Training is more useful when it combines communication practice, reflection on professional assumptions, and institutional support [8,9,10,11,12]. Cultural safety adds an important caution: healthcare professionals should examine the power and effects of their practice rather than decide on behalf of patients that care was culturally appropriate [11].

Social distance measures the closest relationship that a respondent is prepared to accept with a member of another group [13]. It does not measure clinical conduct and does not establish that a respondent will discriminate. It does, however, show how social inclusion is graded. This distinction is relevant to nursing because professional obligations operate within a wider social environment. Nurses may uphold equal treatment while still carrying assumptions about national belonging, cultural difference, or competition for resources.

Intergroup contact theory proposes that contact is more likely to reduce prejudice when it occurs under conditions of cooperation, comparable status, common goals, and institutional support [14,15]. Frequency alone may therefore be a weak indicator. A rushed encounter without interpretation support is different from sustained, respectful contact in which communication is possible. Identity and threat variables may work in the opposite direction. Exclusive definitions of national membership, ethnocentrism, and perceived competition can preserve distance even when demographic characteristics contribute little.

Croatia offers a useful setting for studying these questions. Public debate has long centred on emigration and workforce loss. More recently, employers have recruited workers from Nepal, India, the Philippines, Bangladesh, and other non-European countries [16,17]. This change has increased everyday contact with populations that were previously less visible in Croatian workplaces and public services. It has also introduced social labels whose meanings may be influenced by familiarity, religion, language, appearance, occupation, class, and media narratives.

Research on migrant-sensitive care in Europe has concentrated mainly on access barriers and migrants’ experiences [5,6,7,18]. Regional studies have shown that social distance and prejudice can also be examined within nursing populations [19,20], but evidence concerning newly arrived labour-migrant groups remains limited. Comparing several labels within the same participants can show whether “migrant worker” is treated as a single category or whether attributed origin changes the response.

### 1.1. Healthcare-Services Framework

Systematic reviews of racial and ethnic bias among healthcare professionals show that provider attitudes can be associated with communication, clinical judgement, treatment recommendations, and patient experience. However, the strength and consistency of these relationships vary across studies [21,22]. Social distance is conceptually distinct from implicit bias, but both bodies of evidence support examining professional attitudes without treating them as direct proof of an individual clinical act.

A European scoping review further indicates that racialised migrants may encounter dismissal, distrust, differential treatment, and barriers to access within healthcare systems [23]. Such findings cannot be transferred directly to the present respondents. They establish, however, why attitudes within a communication-intensive profession warrant investigation alongside organisational conditions and patient-reported experience.

Evidence concerning cultural-competence education also advises caution. Training may improve professional knowledge and perceived competence, but patient and health outcomes have rarely been evaluated and have not shown consistent improvement [24]. Completion of training should therefore not be treated as an outcome in itself; evaluation should examine whether communication, understanding, continuity, safety, and patient experience actually improve.

The World Health Organisation’s competency standards for refugee and migrant health emphasise person-centred communication, respectful practice, collaboration, and attention to organisational and structural barriers [25]. This supports an approach that combines individual reflection with interpreter access, translated information, adequate consultation time, and clear escalation pathways when understanding is uncertain.

Quality healthcare is commonly judged through effectiveness, safety, people-centredness, timeliness, equity, integration, and efficiency [26]. Within that framework, origin-based differences in declared social proximity are relevant as a possible workforce-preparedness signal, but they are not themselves measures of healthcare quality.

We therefore applied a structure–process–outcome logic [27]. The survey measures one aspect of workforce context. If social distance influences care, the relationship must be demonstrated through observable processes such as listening, symptom elicitation, explanation, teach-back, interpreter advocacy, pain assessment, shared decision making, and referral. Any downstream relevance must then be confirmed through patient-reported respect, trust and comprehension, continuity, safety, service use, and health outcomes.

### 1.2. Study Aims and Healthcare Contribution

This study had three linked aims: first, to compare social distance toward five migrant-worker labels among nurses and nursing technicians in Croatia; second, to examine its association with national exclusiveness, ethnocentrism, perceived economic insecurity and status threat, religiosity, travel experience, and intercultural contact; and third, to interpret the findings within a healthcare-quality pathway that separates workforce context from unmeasured care processes and outcomes. Social distance was expected to differ across the five labels (H1). National exclusiveness and ethnocentrism were expected to be associated with greater distance (H2), as were economic insecurity and status threat (H3). Wider intercultural experience and better-quality contact were expected to be associated with lower distance (H4). Religiosity was examined as an exploratory correlate. The healthcare contribution is to identify potentially modifiable targets for workforce development and service safeguards and to specify the direct clinical and patient-level tests required next, not to infer unequal treatment from an attitudinal outcome.

## 2. Materials and Methods

### 2.1. Design, Reporting, and Analytical Role

We conducted a cross-sectional quantitative survey from March to April 2026. The analysis examined social distance toward five migrant-worker labels and its identity-, economic-, and contact-related correlates within a broader questionnaire on attitudes toward migrant workers and migrant patients. For the healthcare-quality interpretation used in this article, social distance was specified as a workforce-context variable. It was not defined as a surrogate outcome for communication quality, clinical decisions, patient experience, continuity, safety, healthcare use, or health outcomes. This restriction governs the analysis and conclusions. The study was reported in accordance with the Strengthening the Reporting of Observational Studies in Epidemiology (STROBE) guidelines for cross-sectional studies. The completed STROBE checklist, indicating the manuscript sections in which each applicable reporting item is addressed, is provided as Supplementary Table S1.

### 2.2. Setting and Recruitment

The questionnaire was administered online because the target population worked across institutions and shifts and because private completion was appropriate for a socially sensitive topic. The link was distributed through educational and professional networks of current and former part-time undergraduate nursing students. Participants could forward the invitation to qualified colleagues. Recruitment was therefore non-probability and included a snowball component. The study was designed to test associations within a professional sample, not to estimate the national prevalence of particular attitudes. Participation was solicited through nursing education and professional networks, with onward forwarding by recipients. Recruitment channel and qualification were therefore distinct concepts: the existence of a forwarded invitation alone did not establish eligibility. The design was a convenience/snowball sample and was not intended to support probability-based estimates for all nurses in Croatia.

### 2.3. Participants and Analysis Sample

Eligible participants were aged 18 years or older, had completed secondary education in nursing, held a licence for independent nursing practice or had previous nursing work experience, and were recruited principally through current or former part-time nursing student networks. These programmes enrol already-qualified nurses and nursing technicians; participants were therefore analysed as a professional nursing sample rather than as students without clinical preparation. Questionnaires were excluded when the respondent lacked a basic nursing qualification, did not complete the survey, or showed a systematic inattentive pattern, such as identical responses across long sequences of unrelated items together with inconsistent demographic entries. The final complete-case dataset comprised 453 respondents. No missing values were imputed. All 453 observations in the analytic dataset contributed to each of the five rated origin outcomes; the study did not recruit separate national-origin cohorts. The information available for the present report does not permit a reliable numerical breakdown of submissions excluded by each screening reason, and such counts are consequently not inferred from the final sample. Similarly, the timing of the inattentive-response rule cannot be verified independently. The study size reflects the usable surveys accrued during the recruitment period rather than a prospective population-representative sample-size calculation.

### 2.4. Questionnaire Development and Pilot Study

The questionnaire covered sociodemographic and professional characteristics, national identity, ethnocentrism, religiosity, economic position, perceived status threat, travel, intercultural contact, and social distance. A separate convenience sample of 100 nurses and nursing technicians completed a pilot version and was not included in the main analysis. Pilot testing assessed clarity, completion time, acceptability of sensitive items, and preliminary internal consistency. Cronbach’s α ranged from 0.79 to 0.88 and McDonald’s ω from 0.80 to 0.89 across the multi-item predictor scales. The pilot consistency ranges describe preliminary performance in a separate sample and must not be interpreted as reliability coefficients estimated in the 453-person analytic sample. No evidence of formal cross-cultural measurement invariance is available for the predictor indices. Accordingly, the construct labels describe operational survey composites and do not establish equivalent interpretation across cultural or linguistic groups.

### 2.5. Social Distance Measure

Social distance was measured with an adapted seven-level Bogardus item [13]. For each label, respondents selected the closest relationship they would accept: close family or partner, close friend, neighbour, co-worker, permanent resident or citizen, temporary visitor, or exclusion from living and working in Croatia. Scores ranged from 1 to 7; lower values indicated greater acceptance of social proximity. Croats were included as the majority reference group. The migrant-worker labels were Filipino, Nepalese, Indian, and Bangladeshi workers and migrants from Arab countries. The total migrant-worker score was the mean of the five migrant-group ratings and excluded the Croatian reference rating. The seven relationship categories were ordered from greatest to least accepted proximity and encoded from 1 (family/partner) to 7 (exclusion). For any one participant, the same five target-group labels were rated; the five-item arithmetic mean was used as a descriptive overall index. Because these are single ordinal responses to distinct social stimuli, internal-consistency estimates are not presented as evidence that the categories form a psychometrically interchangeable reflective scale.

The labels were selected because they were recognisable in Croatian labour-migration discourse at the time of data collection. They are not equivalent types of classification: four refer to nationality, whereas “migrants from Arab countries” refers to a broad transnational category. The latter does not represent a uniform culture, religion, language, class, or migration history. In this study, every label functions as a survey stimulus. A rating describes the respondent’s reaction to that stimulus and must not be read as evidence about the people included under it. Translation, language adaptation, and cultural interpretation should be distinguished from statistical consistency. The questionnaire used origin labels as perceived stimuli in the Croatian social context; those labels cannot be presumed culturally equivalent or interpreted as precise descriptors of heterogeneous populations. Formal translation/back-translation and measurement-invariance testing are not documented by the available study report. These limitations reduce the strength of cross-cultural validity claims.

The response levels are ordered, but the intervals between them are not known to be equal. Means were retained as summaries of group-level patterns and as inputs to the prespecified multivariable models; medians and ranges are reported alongside them. Differences in mean scores should not be interpreted as exact psychological distances between relationship categories.

### 2.6. Predictor Variables

National identity was separated into general national pride and national exclusiveness. The exclusiveness index covered protection of the Croatian way of life, concern about the increasing number of foreign workers, and expectations that people who settle in Croatia should adapt to Croatian cultural patterns. Ethnocentrism represented a broader preference for one’s own national or cultural group. Religiosity combined religious affiliation, frequency of practice, and the subjective importance of religion; because these elements may carry different social meanings, its association with social distance was treated as exploratory. The description of these composites identifies their thematic content rather than furnishing a validated item-level codebook. Source items, anchors, reverse coding and final-sample coefficient alpha or omega are not available in the analysis report, so scores should be interpreted as study-specific indices rather than independently validated instruments. In particular, a composite combining religious affiliation, religious practice and the perceived importance of religion warrants cautious interpretation because its components are conceptually distinct.

Economic measures included monthly net income, perceived standard of living, subjective financial pressure, and perceived threat to the employment or status of domestic workers. Intercultural experience covered travel outside Croatia, longer stays abroad, travel outside Europe, stays in countries where Christianity was not the majority religion, and private or professional contact with people of different cultural, ethnic, or religious backgrounds. Contact frequency and perceived contact quality were analysed separately. Travel, contact frequency, and contact quality should not be considered interchangeable: exposure documents opportunity for contact whereas contact quality is a subjective appraisal. Their estimated associations may reflect residual confounding, reverse directionality, and shared self-report method variance.

### 2.7. Statistical Analysis

Categorical variables are reported as frequencies and percentages. Continuous and ordered variables are described with means and standard deviations as well as medians and ranges where relevant. Because each respondent rated every group, the main comparison used a within-participant repeated-measures analysis restricted to the five migrant-worker labels. The Croatian rating was descriptive and was not included in that test. Pairwise comparisons followed the overall test and used Bonferroni adjustment. Standardised differences are reported to show the magnitude of pairwise contrasts. The repeated-measures ANOVA follows the previously reported analysis of numerically coded ordered categories; its F statistic is not an ordinal-model estimate. The published summary does not establish whether sphericity held or whether a Greenhouse–Geisser or Huynh–Feldt adjustment was applied. Results are therefore interpreted descriptively rather than as fully assumption-verified confirmatory evidence. The aggregate tables are insufficient to recover participant-level ranks for a Friedman test or an ordinal repeated-outcome model. The reported standardised pairwise differences use d_av = |M_1_ − M_2_|/√[(SD_1_^2^ + SD_2_^2^)/2], based on the two condition-specific standard deviations; they are not paired-difference standardised effects (d_z). For the five repeated ratings from 453 respondents, the nominal uncorrected within-participant degrees of freedom are 4 and 1808. The reported F statistic and partial η^2^ are mutually consistent through ηp^2^ = (4 × F)/(4 × F + 1808), giving 0.2647. These quantities describe the reported parametric analysis and do not establish equal spacing between ordinal Bogardus response categories.

Pearson correlations described bivariate associations between the total migrant-worker social-distance score and the predictor indices. Hierarchical linear regression entered sociodemographic and professional variables in the first block, economic variables in the second, identity variables in the third, and travel and contact variables in the fourth. Additional final models were fitted separately for each migrant-worker label. Coefficients are interpreted as adjusted associations, not causal effects. Statistical significance was set at *p* < 0.05, with multiplicity adjustment for the pairwise origin comparisons. Statistical analyses were performed using IBM SPSS statistics (version 32.0; IBM Corp., Armonk, NY, USA) and R statistical software (version 4.6.1; R Foundation for Statistical Computing, Vienna, Austria).

### 2.8. Ethics

The study followed the Declaration of Helsinki and received ethical approval from the FoRRestMind Institute Ethics Committee (FMI No. 001-2026; 1 March 2026). The opening page described the study, the approximate completion time, voluntary participation, withdrawal before questionnaire submission, and scientific use of the data. Continuing to the questionnaire indicated informed consent. The survey collected no names, institutional names, addresses, identification numbers, or IP addresses. It included no patients, health records, interventions, or third-person health data.

## 3. Results

### 3.1. Healthcare Workforce and Migrant-Patient Exposure

The sample was predominantly female (87.0%), with a mean age of 34.8 years (SD 6.7) and 11.2 years (SD 7.1) of nursing experience. Overall, 90.3% were employed in healthcare, including 54.5% in hospitals, 18.3% in primary healthcare or health centres, and 9.3% in emergency medical services. Professional contact with migrant patients was never or rarely for 47.0%, occasional for 35.1%, and frequent or very frequent for 17.9%. Thus, 53.0% reported at least occasional professional contact, although fewer than one in five reported frequent exposure. These figures describe the healthcare setting of the sample; they do not establish the quality or consequences of those encounters. Participant characteristics are presented in Table 1. The four named healthcare workplace categories account for 403 participants (247 + 83 + 42 + 31), whereas healthcare employment is reported for 409. Because the questionnaire table also lists social care (*n* = 24) and outside direct healthcare (*n* = 26), the reported classification does not reveal how the six-person difference was coded. Workplace-specific estimates should not be used to infer mutually exclusive sectors without the underlying coding scheme.

### 3.2. Differences Between Migrant-Worker Labels

Croats, retained only as the majority reference, had the lowest social-distance score. Among the five migrant-worker labels, the ordering was Filipino, Nepalese, Indian, Bangladeshi, and migrants from Arab countries, from lower to higher reported distance (Table 2). The omnibus within-participant comparison was significant and large in magnitude (Table 3). All ten pairwise contrasts remained significant after Bonferroni adjustment (Table 4). H1 was supported.

### 3.3. Bivariate Associations

The total migrant-worker score correlated most strongly with national exclusiveness (r = 0.58), ethnocentrism (r = 0.52), and economic insecurity and status threat (r = 0.44). Better contact quality (r = −0.34) and wider travel and intercultural experience (r = −0.27) were associated with lower distance. Religiosity and Christian cultural identity showed a smaller positive association (r = 0.31). Full bivariate results are presented in Table 5.

### 3.4. Multivariable Models

Pearson correlations describe unadjusted relationships and do not isolate the roles of correlated predictors. Hierarchical increments in explained variance depend on the order of variable entry and should not be interpreted as unique causal contributions.

Explained variance increased from 4% in the sociodemographic and professional block to 49% in the full model. National exclusiveness remained the strongest positive correlate, followed by economic insecurity, status threat, and ethnocentrism. Contact quality and wider travel and intercultural experience retained inverse associations. These findings support H2–H4. Religiosity retained a smaller coefficient in the full model and was most clearly associated with the Arab-country label in the separate models. The hierarchical model is presented in Table 6, and the label-specific models are shown in Table 7. In Table 7, the five model columns correspond to separate dependent ratings supplied by the same respondents, not to five nationality-based participant subgroups. Accordingly, the labels denote survey stimuli rather than subgroup membership.

The incremental R^2^ estimates in Table 6 are conditional on block-entry order and should not be interpreted as causal effects.

## 4. Discussion

### 4.1. Principal Findings in the Healthcare-Workforce Context

The origin-specific analyses use five ratings contributed by each respondent, rather than nationality-defined strata of nurses. Comparisons across model coefficients are therefore descriptive and do not themselves test differences between coefficients.

The same nurses and nursing technicians did not respond uniformly to the five migrant-worker labels. The within-participant effect was large, every pairwise comparison remained significant after correction, and the ordering extended from lower distance toward Filipino workers to higher distance toward the broad Arab-country label. H1 was supported. The result demonstrates origin-based differentiation within a professional group responsible for repeated communication and continuity of care; it does not demonstrate origin-based differences in care.

The gradient is relevant to service preparedness because a generic category such as “migrant patient” can conceal different expectations attached to national, regional, linguistic, or religious cues. The questionnaire did not identify which meanings participants assigned to each label. Familiarity, English-language use, occupation, religion, appearance, media coverage, and geopolitical narratives remain plausible but untested explanations. The Arab-country category is especially heterogeneous and must be interpreted only as a broad survey stimulus, not as a description of any population.

National exclusiveness and ethnocentrism remained independently associated with greater distance, supporting H2, while economic insecurity and status threat supported H3. Demographic and professional variables explained comparatively little variance. Religiosity showed a smaller and non-uniform association and cannot be used to infer professional behaviour. These patterns favour organisation-wide approaches rather than targeting staff by age, gender, tenure, workplace, or religion.

Better-quality contact and wider intercultural experience were associated with lower distance, supporting H4, and contact quality retained the stronger adjusted coefficient. This is consistent with intergroup contact theory [14,15]. The cross-sectional design does not establish direction, but the finding is operationally relevant: exposure alone may be insufficient when encounters remain rushed, linguistically unsupported, unequal, or conflictual.

### 4.2. From Workforce Context to Testable Care Pathways

Clinical implications require direct evaluation of the proposed healthcare pathway. The first test is whether social distance predicts encounter processes after patient need and clinical complexity are controlled: listening, symptom elicitation, credibility attribution, pain assessment, explanation, teach-back, shared decision making, interpreter use, time allocation, and referral. The second is whether those processes predict patient-reported respect, trust, comprehension, continuity, service use, and safety. Only prospective linkage to such measures, and ultimately to health outcomes, can establish clinical relevance [21,22,23,26,27].

This staged model also locates responsibility at the appropriate levels. Individual reflection may reduce unchecked assumptions, but equitable care cannot depend on individual goodwill. Interpreter access, translated materials, communication protocols, staffing, escalation routes, and audit systems are structural safeguards. Quality improvement should combine these safeguards with direct process and outcome measures rather than treating completion of cultural-competence training as evidence of patient benefit [24,25].

### 4.3. Implications for Nursing Practice and Healthcare Organisations

Practical responses should combine workforce development with service redesign. Matched clinical cases in which symptoms and urgency are identical but origin cues vary can help teams identify inconsistent judgements. Healthcare organisations should standardise interpreter activation, translated information, teach-back, and escalation when understanding is uncertain. Evaluation should include migrant patients’ reports of respect, trust, involvement, and comprehension, alongside process indicators such as interpreter use, documented understanding, completed referrals, complaints, missed follow-up, and safety events.

The social-distance measure is suitable only for anonymous group-level learning and repeated needs assessment. It should not be used to label individual staff, predict a particular clinical act, or support disciplinary decisions. The quality-improvement endpoint is not a lower questionnaire score but demonstrably consistent communication, access, safety, and continuity across patient groups. The proposed evidence-to-action pathway is summarised in Table 8.

### 4.4. Implications for Health-Professional Education

Education should avoid catalogues of supposed cultural traits because they can reproduce the categories they intend to explain. Greater value lies in case-based communication, cultural safety, reflection on professional assumptions, supervised work with interpreters or cultural mediators, and debriefing of difficult encounters. Educational evaluation should move beyond participant satisfaction and knowledge tests to observed communication and patient-relevant outcomes [11,24,25].

### 4.5. Research Agenda

The next study should prospectively link anonymous workforce measures to direct indicators of care. A multilevel design could pair staff measures with standardised-patient or vignette performance, interpreter use, documentation of teach-back, and migrant patients’ experience and follow-up. Repeated measurement before and after an organisational intervention would test whether improved contact conditions reduce social distance and whether any change corresponds to better communication, continuity, and safety. Qualitative interviews with nurses and migrant patients should clarify what the origin labels signify and which service barriers are most consequential.

### 4.6. Strengths and Limitations

The within-participant design isolates variation elicited by the five labels from stable respondent characteristics. The analysis also distinguishes contact quality from contact frequency and wider intercultural experience, reports effect sizes, and provides adjusted and label-specific estimates. Locating social distance at the workforce-context level generates explicit, falsifiable healthcare questions rather than treating an attitude scale as a clinical endpoint.

Important limitations remain. Non-probability online recruitment through part-time education and professional networks prevents national prevalence estimation and may overrepresent nurses engaged in continuing education. The cross-sectional self-report design prevents causal inference and is vulnerable to selection, social-desirability, and common-method bias; no separate social-desirability scale was included. The adapted Bogardus response is ordinal; intervals between categories are not equal, and the total score combines heterogeneous labels. The absence of item-level codebooks, final-sample measurement reliability, ordinal sensitivity tests, sphericity evidence, and regression diagnostics in the available report limits the reproducibility and statistical robustness that can be independently established from these summary results. The workplace-count discrepancy likewise prevents an unambiguous reconstruction of mutually exclusive occupational categories. None of these missing details should be interpreted as evidence that an unreported test was completed or passed. The available tabulated summaries do not establish rank-based robustness, sphericity diagnostics, or residual diagnostics; consequently, inference from the parametric models is interpreted with appropriate restraint.

The study did not measure the meanings attached to individual labels, contact with each specific population, clinical vignettes, observed communication, treatment decisions, interpreter use, patient-reported experience, adherence, continuity, service use, safety events, or health outcomes. The broad Arab-country label is imprecise. Social distance is therefore neither evidence nor a proxy for discriminatory care. Generalisation is limited to associations in this professional sample, and every proposed healthcare pathway requires direct testing.

## 5. Conclusions

Origin-based social distance varied substantially within this Croatian nursing sample. It was associated more strongly with national exclusiveness, ethnocentrism, perceived status threat, contact quality, and intercultural experience than with basic demographic or professional characteristics. Because more than half of respondents reported at least occasional professional contact with migrant patients, the findings concern a present workforce and service context. Their defensible implication is the need for organisation-wide safeguards and direct monitoring of communication, respect, comprehension, continuity, and safety. The study does not show that social distance caused unequal care or poorer health. It identifies a potentially modifiable upstream context and defines how future research should test whether, when, and through which care processes that context becomes clinically consequential.

## Figures and Tables

**Table 1:** Selected characteristics of participants (N = 453).

Characteristic	*n*	%
Gender: women	394	87.0
Gender: men	59	13.0
Age 25–29 years	96	21.2
Age 30–34 years	128	28.3
Age 35–39 years	112	24.7
Age 40–44 years	71	15.7
Age 45–50 years	46	10.2
Employed in healthcare	409	90.3
Hospital workplace	247	54.5
Primary healthcare or health centre	83	18.3
Emergency medical service	42	9.3
Private healthcare institution	31	6.8
Social care institution	24	5.3
Outside direct healthcare system	26	5.7
Private intercultural contact: never or rarely	122	26.9
Private intercultural contact: occasionally	167	36.9
Private intercultural contact: often or very often	164	36.2
Professional contact with migrant patients: never or rarely	213	47.0
Professional contact with migrant patients: occasionally	159	35.1
Professional contact with migrant patients: often or very often	81	17.9

Note. Percentages use the full sample (N = 453) and may differ slightly due to rounding. Mean age: 34.8 years (SD 6.7; range 25–50); mean experience: 11.2 years (SD 7.1; range 1–30). The healthcare-employment figure and the individually listed workplace categories are different summary variables; the four named healthcare settings total 403, compared with 409 respondents who reported healthcare employment. The original categories do not account for the difference, which is not allocated to a specific workplace.

**Table 2:** Social-distance ratings by group label.

Group Label	Mean	SD	Median	Range	95% CI
Croats as majority reference	1.42	0.66	1	1–5	1.36–1.48
Filipino workers	2.74	1.18	3	1–7	2.63–2.85
Nepalese workers	3.18	1.22	3	1–7	3.07–3.29
Indian workers	3.76	1.31	4	1–7	3.64–3.88
Bangladeshi workers	3.93	1.34	4	1–7	3.81–4.05
Migrants from Arab countries	4.21	1.39	4	1–7	4.08–4.34

Note. Scores range from 1 to 7. Lower scores indicate willingness to accept a closer relationship. The Croatian reference was excluded from the total migrant-worker score and from the main repeated-measures test. CI, confidence interval.

**Table 3:** Repeated-measures comparison across the five migrant-worker labels.

Within-Participant Effect	F	df	*p*	Partial η^2^
Migrant-worker label	162.75	4, 1808	<0.001	0.265

Note. Five repeated ratings from each of 453 respondents were analysed (five origin labels; 2265 ratings). Croats were retained as a descriptive majority reference and excluded from this comparison. The stated degrees of freedom correspond to the conventional uncorrected repeated-measures ANOVA. Partial η^2^ is consistent with the reported F statistic and degrees of freedom (0.265 after rounding).

**Table 4:** Bonferroni-adjusted pairwise comparisons.

Comparison	Mean Difference	SE	*p*	Cohen d
Filipino–Nepalese	−0.44	0.06	<0.001	0.37
Filipino–Indian	−1.02	0.07	<0.001	0.82
Filipino–Bangladeshi	−1.19	0.07	<0.001	0.94
Filipino–Arab-country label	−1.47	0.08	<0.001	1.14
Nepalese–Indian	−0.58	0.06	<0.001	0.46
Nepalese–Bangladeshi	−0.75	0.06	<0.001	0.58
Nepalese–Arab-country label	−1.03	0.07	<0.001	0.78
Indian–Bangladeshi	−0.17	0.04	0.001	0.13
Indian–Arab-country label	−0.45	0.05	<0.001	0.33
Bangladeshi–Arab-country label	−0.28	0.05	<0.001	0.20

Note. Negative mean differences indicate lower social distance toward the first label; SE, standard error. The reported Cohen’s d estimates correspond to rounding, to the absolute mean contrast divided by the root mean square of the two marginal standard deviations, rather than the standard deviation of paired differences.

**Table 5:** Correlations with total migrant-worker social distance.

Variable	r	*p*
National exclusiveness	0.58	<0.001
Ethnocentrism	0.52	<0.001
Economic insecurity and status threat	0.44	<0.001
Religiosity and Christian cultural identity	0.31	<0.001
Subjective financial pressure	0.29	<0.001
Travel and intercultural experience	−0.27	<0.001
Contact quality	−0.34	<0.001

Note. Pearson correlations are shown. The total score is the mean of the five migrant-worker ratings.

**Table 6:** Hierarchical regression predicting total migrant-worker social distance.

Predictor	Model 1 β	Model 2 β	Model 3 β	Model 4 β
Age	0.09 *	0.06	0.04	0.03
Gender: men	0.07	0.06	0.04	0.03
Work experience	0.08	0.05	0.03	0.02
Hospital employment	0.05	0.04	0.03	0.02
Subjective financial pressure		0.18 ***	0.11 **	0.09 *
Economic insecurity and status threat		0.36 ***	0.25 ***	0.21 ***
Ethnocentrism			0.24 ***	0.20 ***
National exclusiveness			0.33 ***	0.29 ***
Religiosity and Christian cultural identity			0.12 **	0.10 *
Travel and intercultural experience				−0.13 **
Contact quality				−0.19 ***
R^2^	0.04	0.23	0.43	0.49
Adjusted R^2^	0.03	0.22	0.42	0.48
ΔR^2^	0.04 *	0.19 ***	0.20 ***	0.06 ***

Note. Standardised coefficients are shown. * *p* < 0.05; ** *p* < 0.01; *** *p* < 0.001.

**Table 7:** Final regression models for individual migrant-worker labels.

Predictor	Filipino β (N = 453)	Nepalese β (N = 453)	Indian β (N = 453)	Bangladeshi β (N = 453)	Arab-Country β (N = 453)
Subjective financial pressure	0.05	0.07	0.10 *	0.11 *	0.09 *
Economic insecurity and status threat	0.14 **	0.18 ***	0.23 ***	0.25 ***	0.22 ***
Ethnocentrism	0.13 **	0.17 ***	0.21 ***	0.22 ***	0.24 ***
National exclusiveness	0.18 ***	0.24 ***	0.31 ***	0.32 ***	0.36 ***
Religiosity and Christian cultural identity	0.04	0.07	0.09 *	0.11 *	0.18 ***
Travel and intercultural experience	−0.12 *	−0.13 **	−0.11 *	−0.10 *	−0.08
Contact quality	−0.16 ***	−0.18 ***	−0.19 ***	−0.17 ***	−0.13 **
Adjusted R^2^	0.24	0.32	0.43	0.45	0.48

Note. Five separate outcomes were modelled using the same 453 respondents, each of whom rated all five labels; the columns are not subsamples of respondents. Models were also adjusted for age, gender, and work experience. Higher scores indicate greater social distance. The final column refers to the broad label “migrants from Arab countries”. * *p* < 0.05; ** *p* < 0.01; *** *p* < 0.001.

**Table 8:** Evidence-to-action pathway for migrant-sensitive healthcare.

Level	What the Study Establishes	What Was Not Measured	Possible Response	Direct Evaluation
Workforce context	Origin labels elicited different levels of social distance; identity, threat, and contact variables were independently associated with distance.	Clinical conduct and patient outcomes.	Anonymous group-level needs assessment and matched clinical cases.	Repeated workforce measures linked to independently assessed practice indicators.
Encounter process	No direct inference can be made from the survey.	Listening, explanation, teach-back, pain assessment, interpreter advocacy, time allocation, and referrals.	Interpreter protocols, translated information, teach-back, adequate time, and escalation pathways.	Observation, standardised patients, documentation review, and patient-reported communication.
Patient experience and continuity	No patient data were collected.	Respect, trust, comprehension, complaints, completed referrals, missed appointments, and follow-up.	Patient feedback, navigation support, and continuity monitoring.	Stratified patient-experience and service-continuity indicators.
Safety and health outcomes	The design provides no evidence of downstream effects.	Adherence, avoidable deterioration, emergency use, complications, and other health outcomes.	Prospective linkage of workforce, process, patient, and service data.	Safety events, service use, adherence, and clinically appropriate health outcomes.

Note. Only the workforce-context row contains outcomes measured in this study. Encounter processes, patient and service outcomes, and health outcomes are proposed validation targets, not observed consequences.

## Data Availability

The de-identified data supporting the findings are available from the corresponding author upon reasonable request, subject to the conditions of the ethics approval and protection of participant confidentiality.

## Supplementary Materials

The following supporting information can be downloaded at https://www.mdpi.com/article/10.3390/healthcare14213581/s1, Table S1: STROBE Checklist for Cross-Sectional Studies.
