# Supplementary material for: Origin-Based Social Distance Among Nurses in Croatia: Implications for Migrant-Sensitive Healthcare Delivery
Source: Healthcare (Basel). 2026 Oct 23;14(21):3581. doi: 10.3390/healthcare14213581 (PMC13763029; doi:10.3390/healthcare14213581)
Supplement: Supplementary file 1 [file healthcare-14-03581-s001.zip › healthcare-4599594-supplementary.pdf]

Table S1: STROBE Checklist for Cross-Sectional Studies

Manuscript: Origin-Based Social Distance among Nurses in Croatia: Implications for Migrant-Sensitive Healthcare Delivery

Study design: Cross-sectional survey

Completed STROBE checklist with revised section and page locations

Item	Recommendation	Location in manuscript
1a	Title and abstract	Title, p. 1. The title identifies the nursing workforce, social-distance outcome, and migrant-sensitive healthcare-delivery context.
1b	Title and abstract	Abstract, p. 1. The abstract reports the cross-sectional design, N = 453, principal estimates, workforce interpretation, and the absence of measured clinical or patient outcomes.
2	Background and rationale	Introduction, Sections 1 and 1.1, pp. 1–2. Migrant-sensitive care, healthcare-quality domains, relevant evidence, and the structure–process–outcome rationale are described.
3	Objectives	Section 1.2, pp. 2–3. Three linked aims, four hypotheses, the healthcare contribution, and the required direct tests of clinical relevance are stated.
4	Study design	Section 2.1, p. 3. Cross-sectional design and the analytical role of social distance as a workforce-context variable are stated.
5	Setting	Sections 2.1–2.2, p. 3. Data-collection period, online setting, and professional recruitment networks are described.
6a	Participants	Section 2.3, p. 3. Eligibility, recruitment route, exclusions, and final complete-case sample are reported.
6b	Matched studies	Not applicable; this was not a matched study.
7	Variables	Sections 2.5–2.6, pp. 3–4. Outcome, predictors, labels, scores, and healthcare variables are defined.
8	Data sources and measurement	Sections 2.4–2.6, pp. 3–4. Questionnaire development, pilot testing, adapted Bogardus measure, and predictor measurement are described.
9	Bias	Sections 2.2–2.3, p. 3, and Section 4.6, pp. 9–10. Selection, self-report, social-desirability, common-method, ordinal-measure, label, and outcome-measurement biases are addressed.
10	Study size	Section 2.3, p. 3. The final complete-case sample comprised 453 respondents; the non-probability design was intended for association testing rather than prevalence estimation.
11	Quantitative variables	Sections 2.5–2.7, pp. 3–4. Scoring, ordered response categories, summary measures, and modelling decisions are explained.
12a	Statistical methods	Section 2.7, p. 4. Repeated-measures analysis, multiplicity adjustment, correlations, hierarchical regression, effect estimates, and significance threshold are reported.
12b	Subgroups and interactions	Section 2.7, p. 4, and Table 7, p. 7. Separate adjusted models for each migrant-worker label are reported.
12c	Missing data	Section 2.3, p. 3. The final analysis used complete cases; no missing values were imputed.
12d	Sampling strategy	Sections 2.2–2.3, p. 3. Non-probability and snowball recruitment are described, with corresponding limits on prevalence inference.
12e	Sensitivity analyses	No separate sensitivity analysis was reported.

Item	Recommendation	Location in manuscript
13a	Participant flow	Section 2.3, p. 3. The final analysis sample of N = 453 is reported.
13b	Reasons for non-participation	Not available because anonymous open-link recruitment did not permit enumeration of all persons who received or declined the invitation.
13c	Flow diagram	A flow diagram was not included because individual invitations and non-response could not be enumerated.
14a	Descriptive data	Section 3.1 and Table 1, pp. 4–5. Demographic and professional characteristics, healthcare employment, setting, and migrant-patient contact are reported.
14b	Missing data by variable	Section 2.3, p. 3. No missing values were present in the final complete-case dataset.
14c	Follow-up time	Not applicable to this cross-sectional survey.
15	Outcome data	Section 3.2 and Tables 2–4, pp. 5–6. Social-distance ratings, omnibus repeated-measures result, and pairwise comparisons are reported.
16a	Main results	Sections 3.3–3.4 and Tables 5–7, pp. 6–7. Bivariate associations, hierarchical models, adjusted coefficients, model fit, and label-specific results are reported.
16b	Category boundaries	Section 2.5, pp. 3–4, and notes to Tables 2–4, pp. 5–6. Category boundaries, direction of scoring, and interpretation of differences are stated.
16c	Translation of relative risk	Not applicable; the study did not estimate relative risks.
17	Other analyses	Table 7, p. 7. Additional label-specific adjusted regression models are reported.
18	Key results	Sections 4.1–4.3 and Table 8, pp. 7–9. Key findings are interpreted at the workforce-context level and linked to testable care-process and outcome pathways.
19	Limitations	Section 4.6, pp. 9–10. Sampling, design, measurement, construct, label, generalisability, and absent healthcare-outcome data are discussed.
20	Interpretation	Sections 4.1–4.5 and Table 8, pp. 7–9, and Conclusions, p. 10. Interpretation is bounded by the results and distinguishes measured workforce context from unmeasured clinical and patient outcomes.
21	Generalisability	Sections 2.2–2.3, p. 3, and Section 4.6, pp. 9–10. Generalisability is limited to associations in this professional sample and not extended to national prevalence, clinical conduct, or patient outcomes.
22	Funding	Funding statement, p. 10. The research received no external funding.
